# Improved chemical text mining of patents using infinite dictionaries, translation and automatic spelling correction

**DOI:** 10.1186/1758-2946-3-S1-O16

**Published:** 2011-04-19

**Authors:** Roger A Sayle, Plamen Petrov, Jon Winter, Sorel Muresan

**Affiliations:** 1 NextMove Software, Santa Fe, New Mexico, 87501, USA; 2 DECS, AstraZeneca, Molndal, Sweden; 3 CIRA, AstraZeneca, Alderley Park, Cheshire, UK

## 

The text mining of patents and patent applications for chemical structures of interest to medicinal chemists poses a number of unique challenges not encountered in other fields of text analytics. Traditional text mining relies on the co-occurrence of common terms between documents to provide similarity measures that can be used to cluster and rank related documents. The more words shared between two documents, the more similar they are, and the greater the probability that they discuss the same topic. By contrast, in pharmaceutical “composition of matter” patents the novel and unique chemical entities are far more significant than those that can be found elsewhere. Although the text of a pharmaceutical patent may explicitly name thousands of individual compounds, and via generic Markush structures claim an infinite number, the role of these patents is to protect the intellectual property of only one or perhaps two drug candidates.

In this work, we present an analysis of the “quality not quantity” of structures extracted by automatic Chemical Named Entity Recognition (CNER) methods both on a small hand-curated benchmark set [1] and a large-scale analysis of a comprehensive database of 12 million patents [2,3]. Our results show the limited value of traditional lexicon/dictionary based approaches in extracting “key” compounds and that the major impediment is not the performance of the name-to-structure software used, but the high rate of OCR errors, typos and lexicographic problems found in patent-office data feeds. To address this problem, novel algorithms for automatic chemical spelling correction have been developed, that take advantage of the grammar used in IUPAC-like nomenclature. This forms a preprocessing pass, independent of the name-to-structure software used, and is shown to greatly improve results in our study.

## References

[B1] Hattori K Wakabayashi H Tamaki K Predicting Key Example Compounds in Competitor’s Patent Applications using Structural Information Alone J Chem Inf Model 2008 48 135 142 10.1021/ci7002686 18177028

[B2] Rhodes J Boyer S Kreulen J Chen Y Ordonez P Mining Patents using Molecular Similarity Search Pac Symp Biocomput 2007 12 304 315 full_text 17990501

[B3] Suriyawongkul I Southan C Muresan S The Cinderella of Biological Data Integration: Addressing the Challenges of Entity and Relationship Mining from Patent Sources Data Integration in the Life Sciences 2010 6254 Springer

[B4] Sayle R Foreign Language Translation of Chemical Nomenclature by Computer J Chem Inf Model 2009 49 519 530 10.1021/ci800243w 19239237 PMC2659868

